# Fabrication and Characterization of Line-by-Line Inscribed Tilted Fiber Bragg Gratings Using Femtosecond Laser

**DOI:** 10.3390/s21186237

**Published:** 2021-09-17

**Authors:** Hongye Li, Xiaofan Zhao, Binyu Rao, Meng Wang, Baiyi Wu, Zefeng Wang

**Affiliations:** 1 College of Advanced Interdisciplinary Studies, National University of Defense Technology, Changsha 410073, China; lihongye@nudt.edu.cn (H.L.); zhaoxiaofan_zxf@nudt.edu.cn (X.Z.); raobinyu@nudt.edu.cn (B.R.); wangmeng@nudt.edu.cn (M.W.); wubaiyi@nudt.edu.cn (B.W.); 2 State Key Laboratory of Pulsed Power Laser Technology, Changsha 410073, China; 3 Hunan Provincial Key Laboratory of High Energy Laser Technology, Changsha 410073, China

**Keywords:** femtosecond laser, direct writing, tilted fiber Bragg gratings

## Abstract

In this paper, we studied the basic characteristics of tilted fiber Bragg gratings (TFBGs), inscribed line-by-line. Experimental results showed that if the TFBGs were located within different planes parallel to the fiber axis, the spectra performed differently. For 2°TFBG, if it was located near the central plane, the Bragg resonance was stronger than ghost mode resonance, and the order reversed if it was located near the boundary between core and cladding. As the tilted angle increased, the range of cladding mode resonance increased. When the tilted angle was larger than 12°, the birefringence effect was observed. Based on the birefringence phenomenon, torsion characteristics were experimentally studied; the sensitivity was about 0.025 dB/degree in the linear variation range. The harmonic order of TFBGs also affected the transmission spectrum. Leaky mode resonance was observed in the 8th order TFBG, and torsion (or polarization) influenced the spectrum of the 8th order TFBG. Our research represented the theory of line-by-line inscribed TFBGs and provided an inscription guidance for TFBGs.

## 1. Introduction

TFBGs can realize the coupling between core mode and cladding modes [1,2]. Owing to the characteristics of cladding mode, TFBGs play an important role in fiber sensing. For example, small angle (<23°) TFBGs, which couple the forward-propagating core mode to backward-propagating cladding modes [1], are largely used in temperature sensing [3] and refractive index sensing [4]. If coated with metal, TFBG-assisted surface plasmon resonance can be excited, with which high sensitivity sensing can be carried out, such as gas/acoustic sensing [5], biological sensing [6], electrochemical activity in supercapacitors [7], and anemometers [8]. In excessively tilted fiber grating (Ex-TFG) (tilted angle > ~70°), the coupling occurs between core mode and cladding modes transmitted in the same direction, which behaves like long period fiber grating. Birefringence characteristics also presents in Ex-TFG. Thus, Ex-TFG is a good candidate for polarization-dependent sensing [9,10]. 45° TFBGs experience high polarization-dependent loss, which provides a method to realize an all-fiber polarizer [11]. In addition to their application in fiber sensing, TFBGs have been applied in fiber lasers. Small angle TFBGs were utilized to suppress the stimulated Brillouin scattering effect in high power fiber lasers [12]. 45° TFBG could play the role of saturable absorber in mode-locked fiber lasers [13,14].

Different methods have been reported to inscribe TFBGs. Ultraviolet exposure is the most common method used [15]. This method can inscribe TFBGs with low insertion loss and high stability. However, the inscription process is complex. Hydrogen loading and annealing are unavoidable during inscription. In the present study, the inscription of Ex-TFG was achieved by adjusting the optical system. Additionally, the resonant wavelength was limited by phase mask (PM). TFBGs could also be made by PM and femtosecond laser radiation [3,16]. Benefits from the femtosecond laser, hydrogen loading, and annealing were avoided, but limitations in the scale and depth of the femtosecond laser focus created difficulties in fiber alignment. To expand the area of refractive index modulation (RIM) and stretch the length of the grating, a complex electrical translation stage was necessary. The resonant wavelength was also limited by PM in this condition. Femtosecond laser plane-by-plane direct writing was an effective method to inscribe TFBG. The tilted angle was controlled by electrical translation stage [17] or the rotating angle of a cylindrical lens in the optical system [18]. The resonance could occur at any wavelength, triggered by adjusting the period of gratings. The birefringence decreased because the RIM was more uniform than TFBGs inscribed with PM [17], but insertion loss increased in the direct writing process. These methods could only fabricate non-localized TFBGs, in which the transmission spectrum was only influenced by the tilted angle. The RIM in the non-localized condition covered the whole core region and the coupling process was relatively stationary [19,20].

Another femtosecond laser direct writing method—namely, line-by-line inscription [21,22]—was also a potential method of fabricating TFBGs. In the year of this study’s writing, Liu et al. proposed TFBGs inscribed line-by-line using femtosecond lasers [23]. Comparing their method with the aforementioned three methods, the TFBGs inscribed line-by-line were highly localized. A benefit of line-by-line inscription was that the insertion loss of TFBGs was low. Additionally, the range of cladding mode resonance was wider than TFBGs inscribed with other methods. Ref. [23] provided preliminary guidance for line-by-line inscription of TFBGs. Further investigation should be carried out to improve this method and the relevant theory of TFBGs.

In this paper, we investigated the fundamental characteristics of TFBGs inscribed line-by-line. The influence of the tilted angle, the line position, and the harmonic order of grating on the transmission spectrum of TFBG were experimentally studied. Some torsional experiments were also carried out to investigate the polarization-dependent effect of TFBGs inscribed line-by-line. Our research works improved the theory of line-by-line inscribed TFBGs and presented guidance for a femtosecond laser direct writing technique.

## 2. Inscription Method

**Figure 1:**
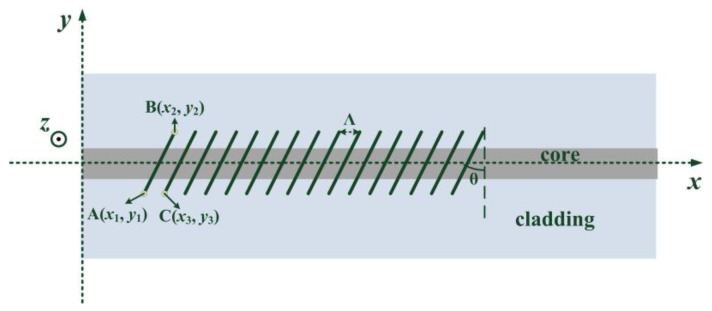
Phase matching condition of the first order harmonic resonance.

Figure 1 shows the schematic of line-by-line inscription of TFBGs. In our experiment, an oil-immersion lens with a magnification of 100× was used to focus the femtosecond laser. The wavelength of the femtosecond laser was 515 nm, the repetition rate was 1 kHz, and the pulse energy after oil-immersion lens was about 36 nJ. The period of grating (Λ) and the tilted angle (θ) were controlled by an electrical 3D translation stage. During inscription, the focus of the femtosecond laser translated from point A to point B. Then, the femtosecond laser was shut off, and the focus of the femtosecond laser moved to the beginning of the next line (point C in Figure 1). After the focus reached point C, the femtosecond laser switched on and began to generate the next line.

The relationship between these three points (A(*x*_1_, *y*_1_), B(*x*_2_, *y*_2_) and C(*x*_3_, *y*_3_)) is as follows:

(1) $$\{\begin{array}{c} y_{1}=-y_{2}=y_{3}\\ x_{2}=2\left|y_{1}\right|\tan\theta +x_{1}\\ x_{3}=x_{1}+\Lambda \end{array}.$$

In order to cover the core diameter, the value of |*y*_1_| is 16 μm. By varying the period of grating (Λ), the resonant wavelength could be tuned. Cladding mode coupling was affected by tilted angle (*θ*). In our experiment, TFBGs were inscribed on single mode fibers (Corning, SMF28). The number of the period was 3000. A sweeping wavelength laser (resolution: 6 pm, and wavelength range: 1503.4–1620nm) was used to record the transmission spectra.

## 3. Effect of TFBG Plane Position on Transmission Spectrum

**Figure 2:**
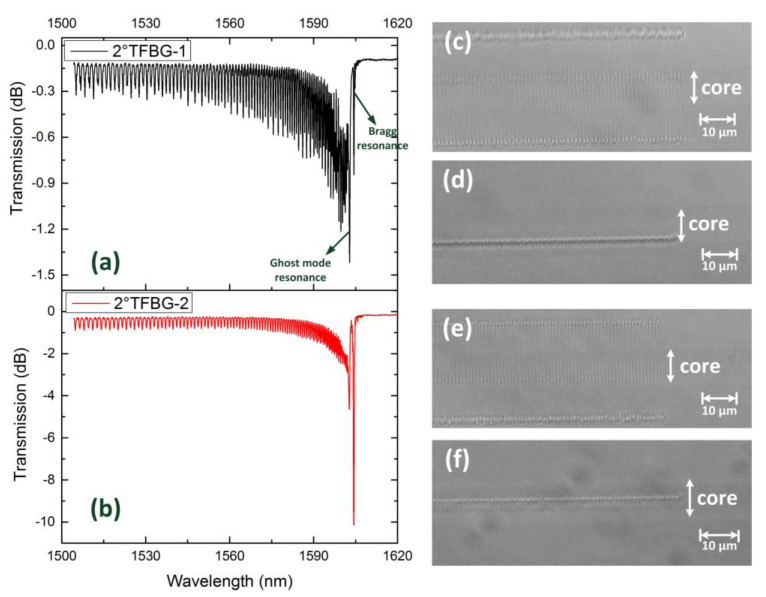
Transmission spectrum of (**a**) 2°TFBG-1 and (**b**) 2°TFBG-2. Microscopy image (100×) of 2°TFBG-1: (**c**) top view and (**d**) side view. Microscopy image (100×) of 2°TFBG-2: (**e**) top view and (**f**) side view.

Figure 2 demonstrates the spectra and microscopies of two 2°TFBGs inscribed on two different planes parallel to the fiber axis. The period of grating was 1.11 μm. Comparing with Figure 2d,f, 2°TFBG-1 was located on a plane near the boundary between core and cladding (off-center inscription in z-direction), and 2°TFBG-2 was located at the central part of the core. That is to say, the overlap between core (or fundamental mode) and RIM of 2°TFBG-2 was stronger than that of 2°TFBG-1. As shown in Figure 2a,b, both the Bragg resonance and the cladding mode resonance of 2°TFBG-1 were weaker than that of 2°TFBG-2 as a result of lower overlap between fundamental mode and RIM. Additionally, the ghost mode resonance of 2°TFBG-1 was stronger than the Bragg resonance; however, the relationship between these two resonances was reversed in 2°TFBG-2. For non-localized inscriptions like the ultraviolet exposure method, the relationship between the Bragg resonance and the ghost mode resonance in TFBG is mainly influenced by tilted angle. As for highly localized inscriptions like femtosecond laser line-by-line inscription, the relationship is also affected by the grating position, and the overall resonant intensity will decrease if the off-center inscription is carried out—which is different from what occurs in non-localized conditions [19,20].

**Figure 3:**
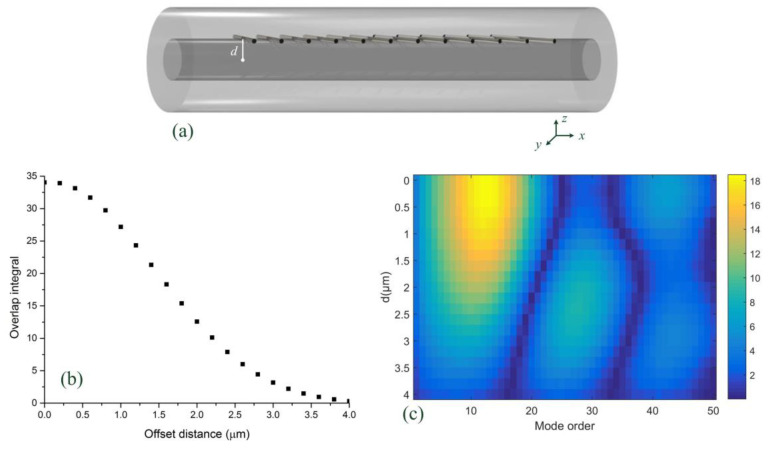
(**a**) Schematic of TFBG with offset distance *d*. (**b**) Overlap integral of fundamental mode versus offset distance. (**c**) Overlap integrals of the first 50 cladding modes under different offset distance.

Figure 3 reveals the calculated results of the overlap integrals under different grating planes. As shown in Figure 3a, the offset distance between the fiber axis and the grating plane is defined as *d*. The overlap integral is read as

(2) $$A=\overset{+\infty }{\underset{-\infty }{\int }}dx(\iint_{S}\Delta \tilde{n}\cdot {\vec{E}}_{01}\cdot {\vec{E}}_{coupled}^{*}\cdot circ\left(r\leq r_{core}\right)drd\varphi )\cdot e^{i\cdot \frac{4\pi }{\Lambda }\cdot x}$$

(3) $$\Delta \tilde{n}=\Delta n\cdot \delta \left(z-d\right)\cdot \delta \left(y-\cot\theta \left(x+m\cdot \Lambda \right)\right)$$

Here, *E*_01_ is the normalized electric field of fundamental mode, *E_coupled_* is the normalized electric field of the mode that fundamental mode couples to, and Δ*n* is the RIM. Additionally, *r_core_* is the radius of the core (in our simulation, this value was 4 μm). The value of *θ* was 2°. The integral along *x* axis was used to extract the Fourier-expansion coefficient of the second order grating. Additionally, n (an integer) represents different lines causing effects on one specific cross section of the fiber core. Although the form of the function of RIM distribution is expressed as impulse function (*δ*) along the *z* axis, we set the modulation region to have a 0.6 μm length along the z axis in simulation. This was done in order to press close to the real inscription in line-by-line condition.

Figure 3b demonstrates the overlap integral of fundamental mode, which decreased with increases in *d*. Figure 3c shows the overlap between fundamental mode and the first 50 cladding modes under different offset distances (*d*). These overlap integrals showed decreasing trends when *d* increased, especially for lower order cladding modes. These phenomena agreed well with what we observed in our experiment.

## 4. Effect of Tilted Angle on Transmission Spectrum

**Figure 4:**
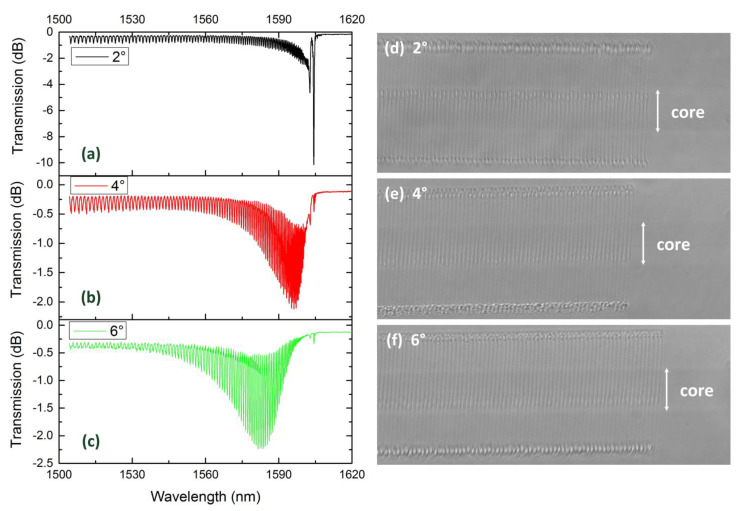
Transmission spectrum of (**a**) 2°TFBG, (**b**) 4°TFBG and (**c**) 6°TFBG, and microscopy image (100×) of (**d**) 2°TFBG, (**e**) 4°TFBG and (**f**) 6°TFBG.

Figure 4 illustrates the transmission spectra and the microscopy images of TFBG with different tilted angles (2°, 4° and 6°). The period of grating was 1.11 μm, which guaranteed the 2nd order resonance at C + L band and the Bragg resonance near 1605nm. Moreover, these three TFBGs were located near the center of the core to avoid weak coupling. As the tilted angle increased, both the Bragg resonance and the ghost mode resonance showed a weakening tendency. Additionally, the envelope of the cladding mode coupling showed an expanding trend and shifted to a shorter wavelength. In other words, a larger tilted angle excited a number of higher order cladding modes and suppressed the coupling of lower order cladding modes.

**Figure 5:**
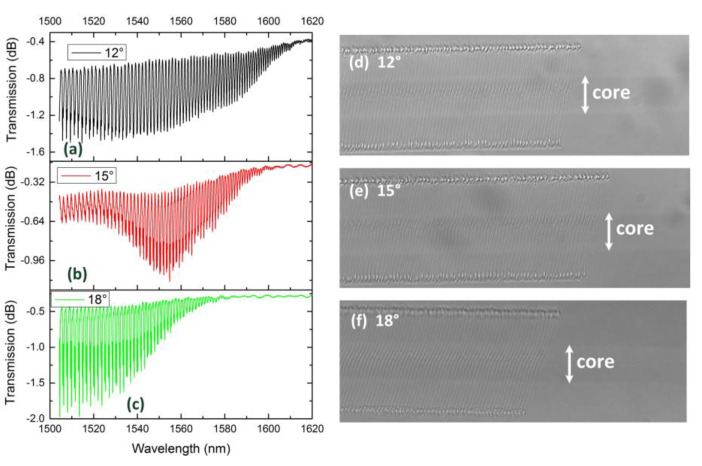
Transmission spectra of (**a**) 12°TFBG, (**b**) 15°TFBG and (**c**) 18°TFBG, and microscopy image (100×) of (**d**) 12°TFBG, (**e**) 15°TFBG and (**f**) 18°TFBG.

Figure 5 performs the transmission spectrum and microscopy image of 12°TFBG, 15°TFBG and 18°TFBG. Considering the limited wavelength range of sweeping wavelength laser, the period of grating is set as 1.13 μm. Except the similar phenomena observed in Figure 3, birefringence is clearly seen on the spectra. Dual dips phenomenon occurs within several resonance wavelengths. Comparing with Figure 4a–c, the envelop of cladding mode coupling becomes blurred. These phenomena are polarization-dependent.

**Figure 6:**
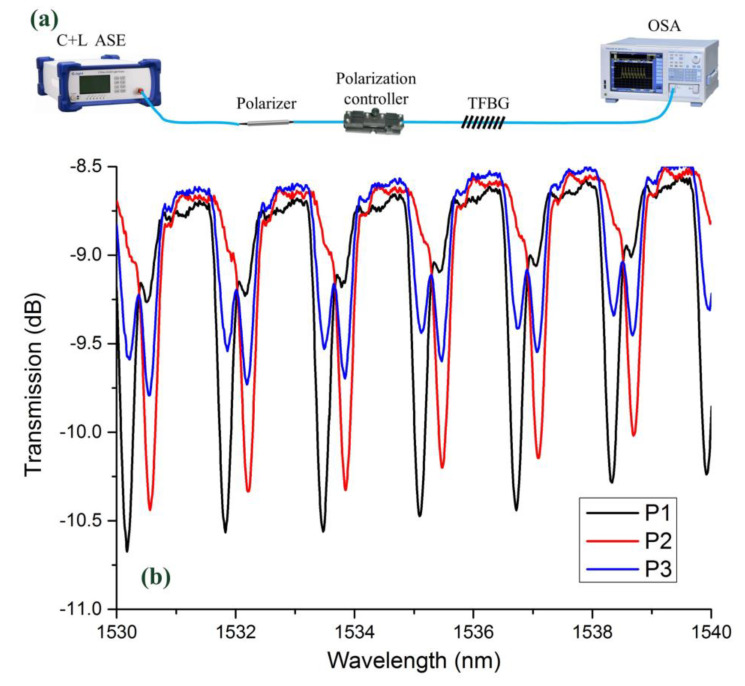
(**a**) Schematic of polarization-dependent spectrum measurement system. (**b**) Spectra of 18°TFBG under different polarization states.

To test the polarization-dependent characteristics, the spectra of 18°TFBG (under different polarization states) were measured. The schematic of spectrum measurement system is illustrated in Figure 6a. After passing through a polarizer, light output from C + L ASE source became linearly polarized. Then, a polarization controller was utilized to adjust the polarization state. Finally, light with different polarization states passed through the TFBG and arrived at an optical spectrum analyzer (OSA). Figure 6b shows the spectra of 18°TFBG under different polarization states. It was obvious that the polarization state had a large impact on the transmission spectrum. Either of the resonant dips could be totally suppressed by tuning the polarization state of the input light.

**Figure 7:**
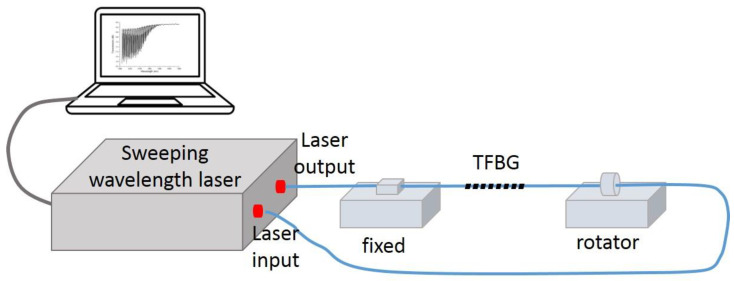
Schematic setup of torsional detection.

We also investigated the torsional effects of 18°TFBG. The torsional detection setup is illustrated in Figure 7. The polarized laser output from a sweeping wavelength laser system, and then passed through a TFBG. After that, a power meter (integrated in the sweeping wavelength laser system) was used to record the spectra of TFBG. The TFBG was fixed between a fiber holder and a fiber rotator. By adjusting the rotating angle of the fiber rotator, different torsional stresses were exerted on the TFBG.

**Figure 8:**
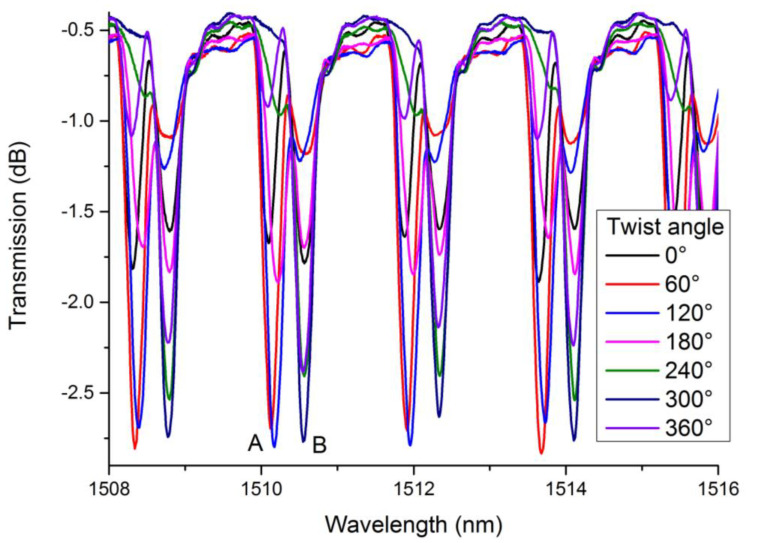
Spectra of 18°TFBG under different twist angles.

Figure 8 shows the spectra of 18°TFBG under different twist angles. For the purposes of our investigation, we only presented the spectra from 1508 nm to 1516 nm. Obviously, twist angles influence the spectrum of 18°TFBG. To quantify our study, we chose two dips (dip A and dip B in Figure 8) near 1510 nm to characterize torsional effects. The resonant wavelengths varied with the twist angle, which could have been induced by stress along the fiber axis in the process of twisting. However, the wavelength shift was extremely small and presented no regular change. The twist-related depth variations of dip A and dip B were much more obvious when compared to wavelength shifts. If one of them deepened, another would weaken.

**Figure 9:**
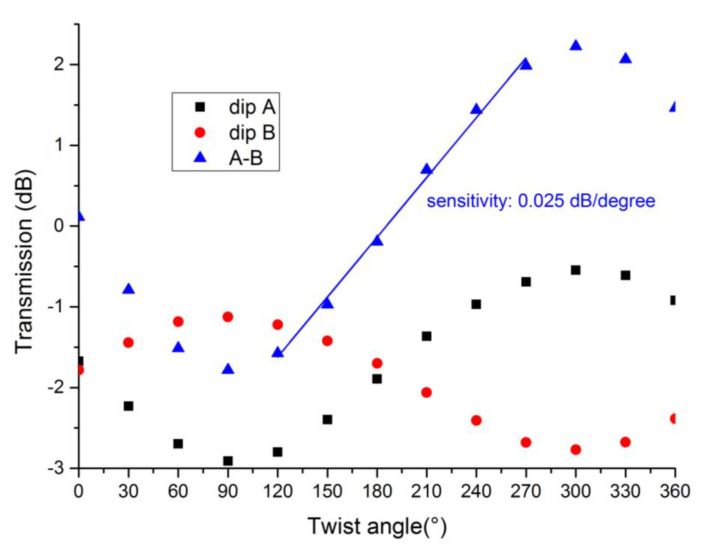
Depth variations of dip A and dip B under different twist angles.

Figure 9 shows the depth variation of dip A and dip B as the twist angle increased with steps of 30 nm. The difference between dip A and dip B was also performed to magnify the twist-related variation. As shown in Figure 9, all the curves took on sine-like shapes. During twist angle increases, the polarization state of input light also rotated; thus, the variation of dip A and dip B also presented a periodical shape. As the blue line shows, the slope (sensitivity) between 120° and 270° was about 0.025 dB/degree. TFBGs with a tilted angle greater than 12° inscribed line-by-line are good candidates in torsional sensing applications. Compared with other kind of torsion sensors, the proposed torsion sensor in our manuscript was highly localized. This provided for the possibility of further integration and would be suitable for narrow area torsion sensing. Moreover, among the benefits of femtosecond laser direct writing, the cladding layer of our sensing head remained intact, a feature which could extend the service life of torsion sensors.

## 5. Effect of Harmonic Order on Transmission Spectrum

**Figure 10:**
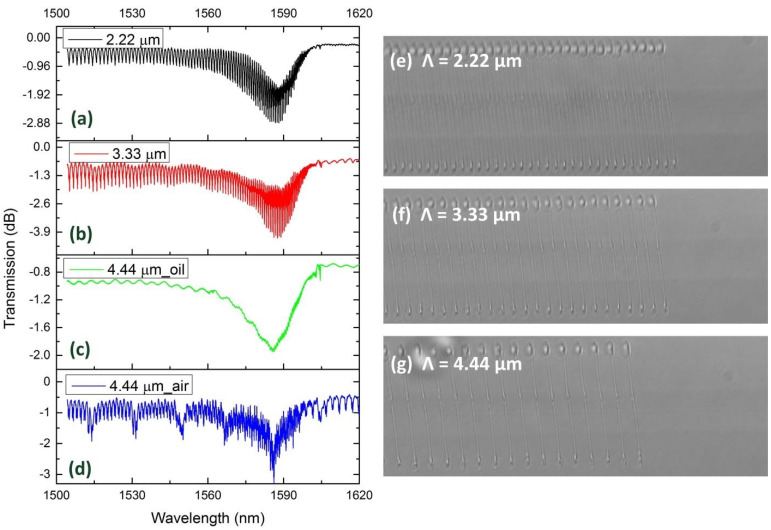
Spectra of 6°TFBGs with different harmonic order: (**a**) Λ = 2.22 μm, (**b**) Λ = 3.33 μm, (**c**) Λ = 4.44 μm (in oil), and (**d**) Λ = 4.44 μm (in air). Microscopy image (100×): (**e**) Λ = 2.22 μm, (**f**) Λ = 3.33 μm, and (**g**) Λ = 4.44 μm.

In addition to the tilted angle, the harmonic order was another factor that influenced the shape of TFBGs. Figure 10 demonstrates the spectra and microscopy images of 6°TFBGs with different harmonic orders. Figure 10a shows the spectrum of the 4th harmonic order 6°TFBG (Λ = 2.22 μm). The characteristics of the 4th harmonic order 6°TFBG were similar to that of the 2nd order (see Figure 4c). However, when the harmonic order increased, the spectrum deteriorated. Figure 10b demonstrates the spectrum of the 6th order 6°TFBG. We observed some tiny fluctuations occurring on the spectrum, which were not present in the 2nd or 4th order 6°TFBG. The spectrum of 8th order 6°TFBG (Figure 10d) was chaotic. Except for the coupling between forward-propagating fundamental modes and backward-propagating cladding modes (the basic characteristics of TFBG), the coupling between the fundamental mode and higher order cladding mode transmitted in the same direction, occurred in the spectrum (the characteristics of LPFGs). Similar phenomena were reported in TFBGs inscribed plane-by-plane, and the authors described this phenomenon as leaky mode resonance (LMR) [24,25]. Additionally, the Bragg resonance and ghost mode resonance disappeared, as shown in Figure 10d. To confirm the existence of the Bragg resonance and ghost mode resonance, we measured the spectrum of 6°TFBG immersed in refractive index matching oil (Figure 10c). Cladding modes were stripped out of the cladding layer, and the Bragg resonance and ghost mode resonance existed in the 8th order 6°TFBG.

**Figure 11:**
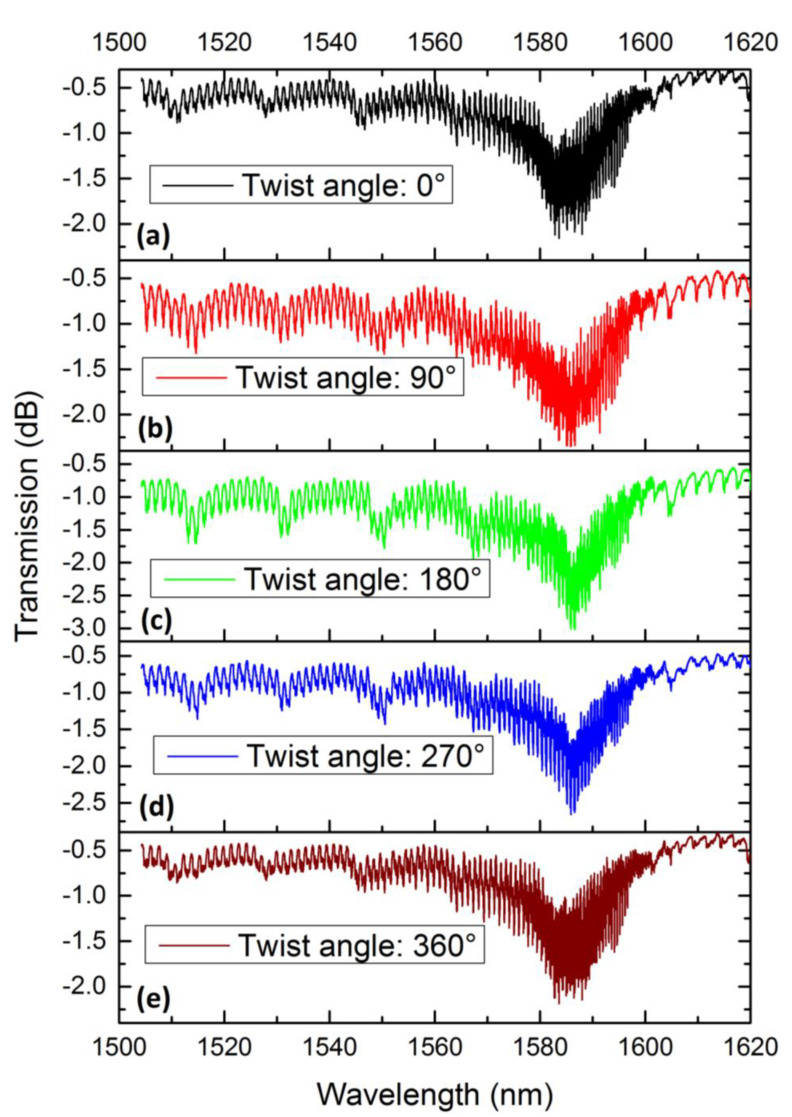
Spectra of the 8th order 6°TFBG under different twist angles: (**a**) 0°, (**b**) 90°, (**c**) 180°, (**d**) 270° and (**e**) 360°.

The torsional effect of the 8th order 6°TFBG was also investigated. The experimental setup was the same as that depicted in Figure 7. Figure 11 perform the spectra under different twist angle. Obviously, the intensity of LMR varied with twist angle, and the resonant wavelength also shifted. To exemplify that, the LMR was relatively weaker when the twist angle was 0° or 360°, the dips reached their deepest points when the twist angle was 180°, and the resonant wavelengths also shifted to longer wavelengths. However, with the impact of the coupling between forward-propagating fundamental mode and backward-propagating cladding modes, we could not simply describe this process by tracking the variation of the resonant wavelengths or the depth of LMR. A more appropriate methodology should be used to study this process.

Correlation is a proper method to consider all phenomena simultaneously. The correlation can be calculated as

(4) $$C_{\alpha }=\frac{\sum_{i=1}^{n}\left(A_{0}[i]\cdot A_{\alpha }[i]\right)}{\sqrt{\sum_{i=1}^{n}A_{0}^{2}[i]}\cdot \sqrt{\sum_{i=1}^{n}A_{\alpha }^{2}[i]}}$$

(5) $$A_{\alpha }[i]=S_{\alpha }[i]-\left(\sum_{i=1}^{n}S_{\alpha }[i]\right)/n$$

**Figure 12:**
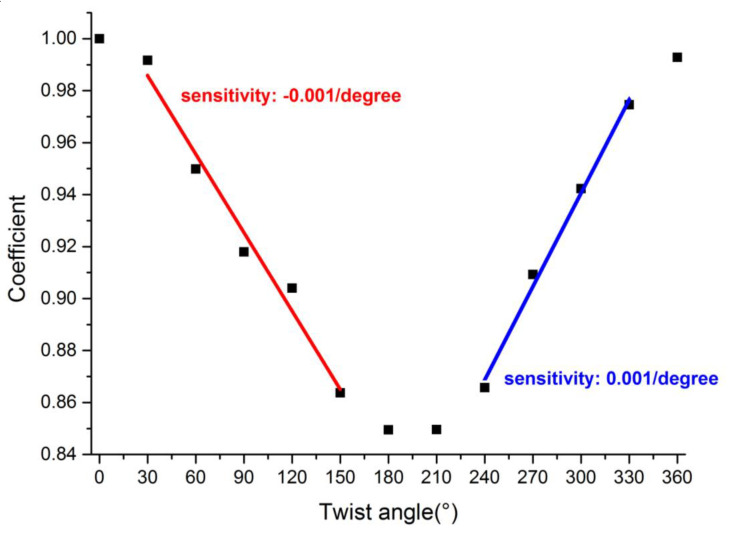
Correlation of spectra under different twist angles.

Here, *S*_α_[*i*] represents the *i*th point on the spectrum of the 8th order 6°TFBG when the twist angle is α. The total number of points on the spectrum was defined as n. Figure 12 illustrates the correlation function verses the twist angle. The whole curve took on a sinusoidal tendency, which indicates that the 8th order 6°TFBG was a polarization-dependent device. The slope (sensitivity) between 30° and 150° was −0.001/degree, and the slope (sensitivity) between 240° and 330° was 0.001/degree. These two ranges were deemed suitable for twist sensing.

## 6. Conclusions

In conclusion, we experimentally studied the characteristics of line-by-line inscribed TFBGs. Studies show that, if the TFBG is located at a different plane parallel to the fiber axis, the transmission spectrum will show large differences. By increasing the tilted angle, the range of cladding mode resonance increased and the minimum value of cladding mode resonance shifted to a shorter wavelength. When the tilted angle was larger than 12°, birefringence effects became obvious. The dual resonant dips of 18°TFBG varied periodically with twist angle and the slope (sensitivity) of the linear variation range was about 0.025 dB/degree, which made these kinds of TFBG a good candidate for twist sensing. Clearly, LMR was observed in the 8th order TFBG, and twist (or polarization state) impacted the spectrum of the 8th order TFBG. By calculating the correlation function of the transmission spectra of the 8th order TFBG under different twist angles, we were able to demodulate the variation tendency, such that we realized twist sensing. TFBG is also a good candidate for refractive index sensing, e.g., biological sensing and chemistry sensing. Outside of their applications in sensing and communication, FBGs with relatively small angles can be applied in stimulated Brillouin scattering suppression in high power fiber lasers. Our research results provide guidance for highly-localized TFBG inscription, which could be meaningful in integrated device fabrication, multichannel sensing, and control of polarization states.
